# Supplementary material for: Pathogenic Germline Mutations of DNA Repair Pathway Components in Early-Onset Sporadic Colorectal Polyp and Cancer Patients
Source: Cancers (Basel). 2020 Nov 28;12(12):3560. doi: 10.3390/cancers12123560 (PMC7761471; doi:10.3390/cancers12123560)
Supplement: Supplementary file 1 [file cancers-12-03560-s001.pdf]

Supplementary Materials

Pathogenic Germline Mutations of DNA Repair Pathway Components in Early-onset Sporadic Colorectal Polyp and Cancer Patients

Pi-Yueh Chang [1,2], Shih-Cheng Chang [1,2], Mei-Chia Wang [1,2], Jinn-Shiun Chen [3], Wen-Sy Tsai [3], Jeng-Fu You [3], Chia-Chun Chen [4], Hsiu-Ling Liu [1] and Jy-Ming Chiang [3,*]

[1] Department of Laboratory Medicine, Chang Gung Memorial Hospital, No. 5 Fu-Shing St. Kweishan, Taoyuan 33305, Taiwan ; changpy@adm.cgmh.org.tw (P.-Y.C.); changsc137@cgmh.org.tw (S.-C.C.); ottermika@cgmh.org.tw (M.-C.W.); s48633001@cgmh.org.tw (H.-J.L.)
[2] Department of Medical Biotechnology and Laboratory Science, Chang Gung University, No. 259, Wenhua 1st Rd., Kweishan, Taoyuan 33302, Taiwan
[3] Department of Colorectal Surgery, Chang Gung Memorial Hospital, No. 5 Fu-Shing St. Kweishan, Taoyuan 33305, Taiwan; chenjs@cgmh.org.tw (J.-S.C.); wensyt@gmail.com (W.-S.T.); you3368@cgmh.org.tw (J.-F.Y.)
[4] Molecular Medicine Research Center, Chang Gung University, No. 259, Wenhua 1st Rd., Kweishan, Taoyuan 33302, Taiwan; chenchiachun@gap.cgu.edu.tw (C.-C.C.)
* Correspondence to: jmjiang@cgmh.org.tw(J.-M.C.); Tel: 886-3-2281200 ext. 2101; Fax: 886-3- 3285818

Supplementary Table 1: Detailed clinical and genetic information for the nine mutation carriers

Group	sample ID	Age	Gender	Stage	Gene	Zygosity	Location	NT Change	A.A. Change	Mutation type	ClinVar/VarSome annotation	ExAc_EAS
Normal	2307	39	M		BRCA2	Heterozygous	exon11	c.2652dupA	p.D885Rfs*2	frameshift insertion	Not reported /Pathogenic	
Polyps	1941	41	F		CHEK2	Heterozygous	exon4	c.472delA	p.I158Yfs*9	frameshift deletion	Not reported/ Likely Pathogenic	0.0006
Polyps	1972	49	F		CHEK2	Heterozygous	exon4	c.472delA	p.I158Yfs*9	frameshift deletion	Not reported/ Likely Pathogenic	0.0006
CRC	1657	46	F	IV	BLM	Heterozygous	exon19	c.3566delT	p.F1189Lfs*9	frameshift deletion	Likely pathogenic/ Pathogenic	
CRC	2299	48	M	III	MLH1	Heterozygous	exon10	c.C793T	p.R265C	nonsynonymous SNV	Pathogenic/ Pathogenic	
CRC	2423	46	M	II	MLH1	Heterozygous	exon11	c.C1029A	p.Y343X	stopgain	Not reported/Pathogenic	
CRC	2757	48	F	IV	MLH1	Heterozygous	exon10	c.C793T	p.R265C	nonsynonymous SNV	Pathogenic/ Pathogenic	
					BRCA1	Heterozygous	exon15	c.C4729T	p.Q1577X	stopgain	Pathogenic/ Pathogenic	
CRC	3248	36	M	III	MLH1	Heterozygous	exon11	c.C1029A	p.Y343X	stopgain	Not reported/ Pathogenic	
CRC	SP-0129	38	F	IV	NTHL1	Heterozygous	exon4	c.699_700del	p.S234Rfs*37	frameshift deletion	Not reported/ Pathogenic	

SNV: single-nucleotide variant; MAF in ExAc_EAS: minor allele frequency in the Exome Aggregation Consortium_East Asia

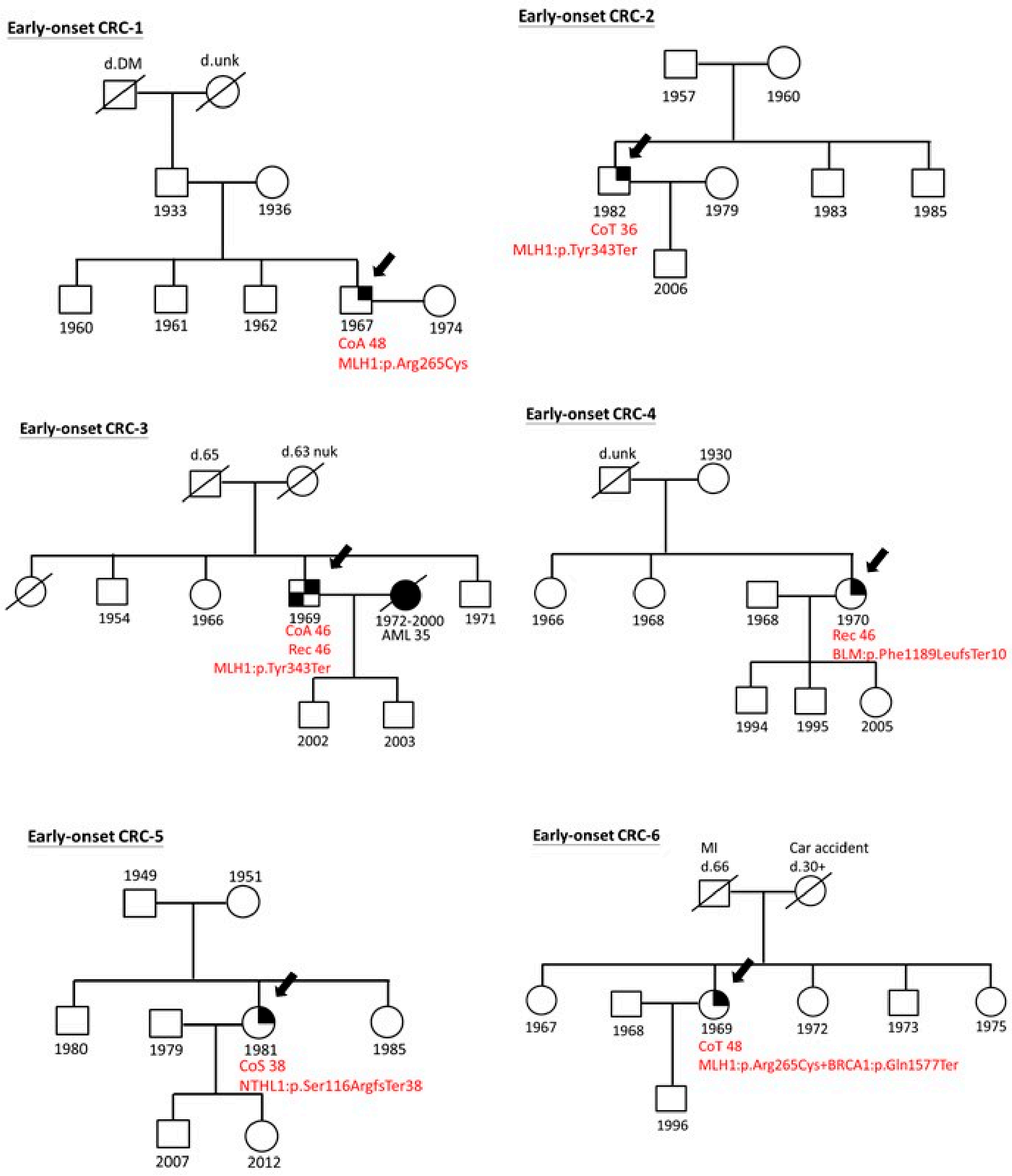

Supplementary Figure 1. Three-generation pedigree trees of six sporadic early-onset CRC proband families. The arrow points out the proband in the family. The number below the square or circle symbol stands for the birth year of the family member. The three letters and two-digit number below the proband represent for the cancer location and the age with cancer diagnosis. For example: CoT 48 indicates the patient got the diagnosis at 48 years old and the cancer was located at the Transverse colon. A: Ascending; S: Sigmoid; Rec: Rectum. Mutations which the proband carried were denoted in the third row.
